# A 23-year-female with plexiform neurofibroma type 1: a rare clinical image

**DOI:** 10.11604/pamj.2024.47.55.42510

**Published:** 2024-02-08

**Authors:** Switi Jawade, Archana Teltumde

**Affiliations:** 1 Department of Obstetrics and Gynaecology Nursing, Smt. Radhikabai Meghe Memorial College of Nursing, Sawangi Meghe Wardha, India,; 2 Datta Meghe Institute of Higher Education and Research, Sawangi Meghe Wardha, India

**Keywords:** Hereditary, plexiform neurofibroma, benign nerve sheath tumour

## Image in medicine

Neurofibromatosis type 1 (NF1) is a hereditary autosomal dominant tumour predisposition condition. This condition is distinguished by benign nerve sheath tumours. Foot NF1-associated neurofibromas are classified as either cutaneous or plexiform. Plexiform neurofibroma of the foot can cause significant functional and cosmetic difficulties. The 23-year-old female patient was admitted to hospital for the treatment of swelling over the lower extremity. The patient had a bike accident many months before and the swelling had been growing slowly. The swelling was painless and the skin was unaffected over months. The skin later become reddish, and patient had increased pain over the lower left foot and difficulty in walking due to the pain. The patient took medical advice. Proper examination revealed that there are multiple plexiform neurofibroma over the leg. Initially, the case was diagnosed as plexiform neurofibroma and incised under local anaesthesia. The initial diagnosis was later overturned and the patient was advised to seek surgical debulking.

**Figure 1:**
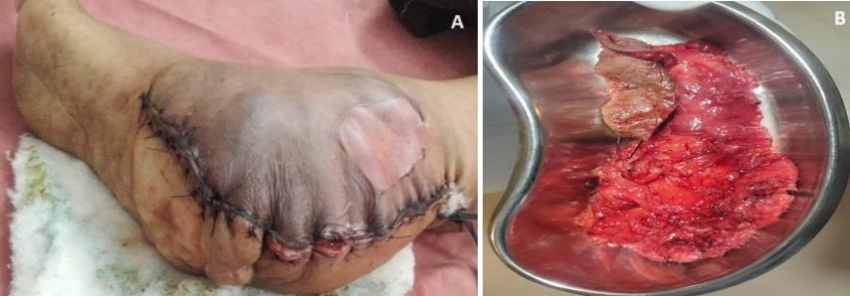
A) surgical debulking; B) plexiform mass

